# Favorable Outcome of Ramsay Hunt Syndrome under Dexamethasone

**DOI:** 10.1155/2012/247598

**Published:** 2012-09-06

**Authors:** Josef Finsterer, Arian Bachtiar, Anton Niedermayr

**Affiliations:** ^1^Krankenanstalt Rudolfstiftung, Postfach 20, 1180 Vienna, Austria; ^2^Department of Otorhinolaryngology, KAR, Juchg. 25, 1030 Vienna, Austria; ^3^Gynecological Department, Krankenanstalt Rudolfstiftung, KAR, Juchg. 25, 1030 Vienna, Austria

## Abstract

A 20-year-old student under chronic stress developed a painful reddish left ear, vesicles on the left ear, severe left-sided peripheral facial-nerve palsy, and hypoesthesia of the left upper lip, after exposure to a ventilator. Ramsay Hunt syndrome was diagnosed. Instead of prednisolone she received dexamethasone (40 mg/d) but nonetheless recovered completely after 12 weeks.

## 1. Introduction

Bell's palsy, also known as idiopathic peripheral facial nerve palsy, accounts for about half of the peripheral facial nerve palsies [1]. Other causes of peripheral facial nerve palsy include trauma, otitis media, mastoiditis, geniculate herpes, tumors along the intracranial nerve roots, diabetes, borreliosis, or sarcoidosis [1]. Ramsay Hunt syndrome (RHS), also known as geniculate herpes or zoster oticus, is characterized by a combination of a peripheral facial nerve palsy with vestibulocochlear symptoms and an erythematous vesicular rash on the ear (zoster oticus) or hard palate [2–4]. Additionally, the trigeminal, glossopharyngeal, vagal, or hypoglossal nerves may be involved. Treatment of choice in the acute phase is steroids in addition to virostatic treatment but it is unknown if the therapeutic effect is dependent on the type of steroids applied. 

## 2. Case Report

We report the case of a 20-year-old Caucasian, nonsmoking female student, height 185 cm, weight 66 kg, who developed pain of the left pinna which is why she attended the otolaryngological ambulatory unit two days later (Table 1). The left pinna was found to be sore and reddish but without vesicles, which is why chondritis was diagnosed and amoxicillin and clavulanic acid prescribed. One day later she awaked with a peripheral facial nerve palsy on the left side and hypoesthesia of the left upper lip. The weeks before onset she had a lot of stress with her studies at the university but there was no fever, no vaccination, or infection. A few days before onset of the palsy she recognised dysesthesias of the parietal skull on the left side. The night before onset of the palsy she was exposed against the ventilator of an air condition for more than an hour. She was regularly taking loratadine. 

Clinical neurologic examination on admission revealed slight hypoesthesia of the left upper lip, moderate peripheral facial nerve palsy, and generally reduced tendon reflexes. The left pinna showed an erythematous rash but no vesicles. Hearing and taste were normal and there was no vertigo, double vision, skew deviation, nystagmus, headache, hyposmia, or ataxia [5]. Blood chemical investigations revealed slight leucopenia but normal C-reactive protein. Ophthalmologic examinations excluded a zoster of the eye. Immediately after admission, she was treated with dexamethasone (40 mg intramuscularly) during two days followed by dexamethasone orally during another six days. Additionally, she received 750 mg acyclovir three times a day intravenously during eight days. For continuous pain periauricularly she received mefenamic acid and paracetamol with success.

At followup three days later she reported some transient vesicles at the left pinna, no longer visible at the visit, lid closure had improved, and there was recovery from the pinna's rash but only marginal voluntary innervation of the frontalis and lower facial muscles. Audiometry was normal. Followup six days after onset of the palsy showed complete lid closure but unchanged innervation of the other facial muscles. She reported neuralgiform pain in the left ear which is why gabapentin was prescribed with success. Eleven days after onset of facial palsy lid closure on the left side was complete. There were slight innervation of the frontal branch and only slight innervation of the left mouth corner. Nerve conduction studies 12 days after onset showed almost equal compound muscle action potential (CMAP) amplitude when recording from the orbicularis oculi muscle but >50% amplitude reduction on the left side when recording from the orbicularis oris muscle. Antibodies against the varicella zoster virus (VZV) were determined three times by means of an ELISA test. IgG antibodies against VZV were positive at all three determinations and IgM antibodies against VZV were negative at the first and positive at the second and third determination, which is why valacyclovir orally was given for seven days after discontinuation of acyclovir. Antibodies against other viruses and *Borrelia burgdorferi* were normal. Follow-up 6 weeks after onset showed further recovery of the palsy, such that it was no longer visible in the absence of emotional reactions. Nerve conduction studies confirmed the improvement such that the CMAP amplitude difference was only reduced to 30%. 

## 3. Discussion

RHS is a polycranial neuritis characterized by damage of motor and sensory neurons, including the facial and the vestibulocochlear nerve and zoster oticus that accounts for approximately 12% of the facial nerve palsies [6, 7]. Accordingly, RHS presents with a combination of a peripheral facial nerve palsy accompanied by vestibulocochlear symptoms and additionally an erythematous vesicular rash of the ear (zoster oticus) or hard palate [2–4]. Some experts, however, indicate that vesicles are no prerequisite to the diagnosis of a RHS [8]. Vestibulocochlear symptoms manifest as otalgia, hypoacusis, tinnitus, or vertigo [9]. In a study on 15 RHS cases involvement of the vestibulocochlear system exclusively manifested as abnormal audiometry due to cochlear or retrocochlear affection [10]. In addition to facial nerve palsy, patients with RHS may also show trigeminal, glossopharyngeal, vagal, or hypoglossal nerve paralysis [4, 11]. RHS is caused by a VZV infection in the ganglion geniculi [4] why PCR for VZV may be positive in the CSF but also in the submandibular saliva, parotid saliva, or tear fluid [12]. RHS is usually treated with prednisolone and acyclovir or valacyclovir within three days of onset [3]. Duration of steroid treatment ranges from 3 days (hydrocortisone) [13] to three weeks. The outcome and prognosis of RHS are usually favorable if treatment with steroids and antiviral agents starts immediately after onset of the clinical manifestations but usually less favorable in Bell's palsy [14]. 

In the presented patient RHS was diagnosed upon the clinical findings with facial palsy, parietal dysesthesia, hypesthesia of the lip, auricular rash, painful pinna, chondritis of the ear, and the positive IgG and IgM antibodies against the VZV virus. Incomplete RHS was diagnosed because of the absence of pronounced vestibulocochlear symptoms, erythematous vesicles on the ear or palate (vesicular rash), and of involvement of cranial nerves IX, X, XI, or XII. Nevertheless, the patient was treated in the same way as the full picture of RHS with steroids and antiviral agents with considerable improvement during the first six weeks. 

The question whether dexamethasone or prednisolone should be favored for the treatment of RHS is unsolved, since there are no studies comparing the effect of both steroids in a double-blind, cross-over trial. Only in a single study oral dexamethasone was compared with prednisone intravenously [8]. In this study there was no difference concerning the outcome and prognosis of RHS among 39 patients with RHS, initially misdiagnosed in 23 cases as Bell's palsy (*n* = 9), sudden sensory hearing loss (*n* = 5), herpes zoster pharyngitis, (*n* = 1), or acute otitis media (*n* = 1) [8]. However, prednisolone has been applied in most of the studies on RHS, which per se does not justify assessing it as more effective than dexamethasone [15]. Since it is unknown if either prednisolone or dexamethasone should be applied in the acute stage, both can be alternatively applied. In a study on 91 patients with RHS it turned out that the combination of acyclovir together with steroids is more favorable concerning the outcome than steroids alone [15]. Low evidence for any treatment is provided by three Cochrane reviews pointing out that there are insufficient data available to confirm the effectiveness of steroids or of antiviral agents for the treatment of RHS [16–18]. Comparison of prednisone and dexamethasone in non-RHS patients, however, suggests that dexamethasone may have a stronger antiedematous effect as compared with prednisone [19]. 

Concerning the contribution of valacyclovir to the recovery of the patient, it has been repeatedly reported that this antiviral agent is effective in RHS if given within three days after onset of symptoms in combination with prednisolone [3]. Despite the frequent application of valacyclovir in RHS, however, it is under debate to which degree the agent contributes to the outcome of RHS patients. No studies are available which compared valacyclovir and steroids, each in monotherapy, concerning their effect in RHS. In a study on 196 patients with Bell's palsy or RHS the outcome was mainly predicted by the initial degree of denervation [20]. In this study recovery was incomplete after one year if initial denervation was >90%, irrespective of the drugs applied [20]. As long as open questions concerning the antiviral treatment of RHS are unsolved, however, RHS patients should receive antiviral agents in addition to dexamethasone or prednisolone.

In conclusion this case shows that RHS partially recovers under combined intramuscular and oral application of dexamethasone within six weeks. 

## 4. Bullet Point Summary

In single cases Dexamethasone may be effective in Ramsay Hunt syndrome (RHS).

In single cases RHS recovers completely under dexamethasone. 

Consider that the trigeminal, glossopharyngeal, vagal, or hypoglossal nerve may be additionally affected in RHS. 

## Figures and Tables

**Table 1:** Time course of the development and resolution of symptoms and signs and duration of treatment.

Time course	Symptoms/signs	Drug treatment
Day 1	Pain of left pinna	None
Day 3	Sore/reddish pinna, chondritis diagnosed Exposure to a ventilator for >1 h	Amoxicillin/clavulanic acid
Day 4 (onset)	Left peripheral facial palsy	Dexamethasone, acyclovir
Day 7	Improvement of lid closure, pinna rash ↓	Dexamethasone, acyclovir
Day 10	Complete lid closure, neuralgia left ear	Dexamethasone, acyclovir, gabapentine
Day 11	Complete lid closure	Dexamethasone, acyclovir stopped
Day 12	Complete lid closure	Starting valacyclovir
Day 15	Slight voluntary contraction of forehead and left mouth corner	Valacyclovir
Day 18	Further improvement of the palsy	Valacyclovir stopped
Day 47	Palsy visible only with mimic contraction	None
Day 85	Complete recovery	None
